# *N*-Ferrocenymethyl-*N*-phenyl­propionamide

**DOI:** 10.1107/S1600536812016303

**Published:** 2012-04-21

**Authors:** Abdelhamid Khelef, Belgacem Terki, Mohammed Sadok Mahboub, Touhami Lanez

**Affiliations:** a VTRS Laboratory, Institute of Sciences and Technology, University of El-Oued, PO Box 789, El-Oued 39000, Algeria; b Department of Chemistry, University of Ouargla, PO Box 511, Ouargla 30000, Algeria

## Abstract

In the title compound, [Fe(C_5_H_5_)(C_15_H_16_NO)], the two cyclo­penta­dienyl (Cp) rings are nearly parallel to each other, forming a dihedral angle of 3.7 (1)°, and adopt a staggered conformation. The amide group is almost perpendicular to the plane of the substituted Cp ring, with a C—N—C—C torsion angle of 101.3 (2)°, and the N and O atoms in the ethanoyl group are coplanar, with a C—N—C—O torsion angle of −0.7 (3)°. Weak C—H⋯O hydrogen bonds link adjacent mol­ecules.

## Related literature

For background to the design and properties of ferrocene derivatives, see: Argyropoulos & Coutouli-Argyropoulou (2002 ▶); Cano *et al.* (1995 ▶); Kelly *et al.* (2007 ▶); Shaabani & Shaghaghi (2010 ▶); Torres *et al.* (2002 ▶). For the synthesis of *N*-ferrocenyl­methyl­aniline, see: Osgerby & Pauson (1961 ▶).
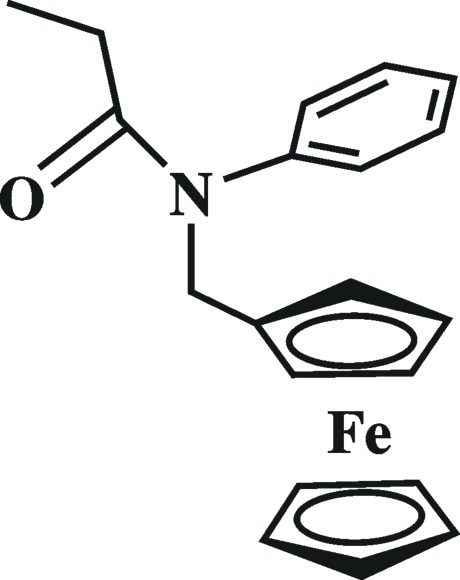


## Experimental

### 

#### Crystal data

[Fe(C_5_H_5_)(C_15_H_16_NO)]
*M* *_r_* = 347.23
Monoclinic, 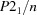

*a* = 13.243 (5) Å
*b* = 7.983 (5) Å
*c* = 15.248 (5) Å
β = 94.873 (5)°
*V* = 1606.2 (13) Å^3^
*Z* = 4
Mo *K*α radiation
μ = 0.94 mm^−1^
*T* = 293 K
0.30 × 0.10 × 0.10 mm

#### Data collection

Nonius KappaCCD diffractometer
Absorption correction: refined from Δ*F* (*DIFABS*; Walker & Stuart, 1983 ▶) *T*_min_ = 0.823, *T*_max_ = 0.991
16518 measured reflections
3670 independent reflections
2747 reflections with *I* > 2σ(*I*)
*R*_int_ = 0.048

#### Refinement

*R*[*F* ^2^ > 2σ(*F* ^2^)] = 0.034
*wR*(*F* ^2^) = 0.094
*S* = 1.02
3670 reflections
209 parameters
H-atom parameters constrained
Δρ_max_ = 0.40 e Å^−3^
Δρ_min_ = −0.38 e Å^−3^

### 

Data collection: *COLLECT* (Nonius, 1998 ▶); cell refinement: *DENZO*/*SCALEPACK* (Otwinowski & Minor, 1997 ▶); data reduction: *DENZO*/*SCALEPACK*; program(s) used to solve structure: *SIR92* (Altomare *et al.*, 1994 ▶); program(s) used to refine structure: *SHELXL97* (Sheldrick, 2008 ▶); molecular graphics: *ORTEP-3* (Farrugia, 1997 ▶); software used to prepare material for publication: *WinGX* (Farrugia, 1999 ▶).

## Supplementary Material

Crystal structure: contains datablock(s) global, I. DOI: 10.1107/S1600536812016303/hy2528sup1.cif

Structure factors: contains datablock(s) I. DOI: 10.1107/S1600536812016303/hy2528Isup2.hkl

Additional supplementary materials:  crystallographic information; 3D view; checkCIF report

## Figures and Tables

**Table 1:** Hydrogen-bond geometry (Å, °)

*D*—H⋯*A*	*D*—H	H⋯*A*	*D*⋯*A*	*D*—H⋯*A*
C4—H4⋯O^i^	1.07	2.50	3.292 (4)	130

Symmetry code: (i) 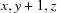.

**Fig. 1.**
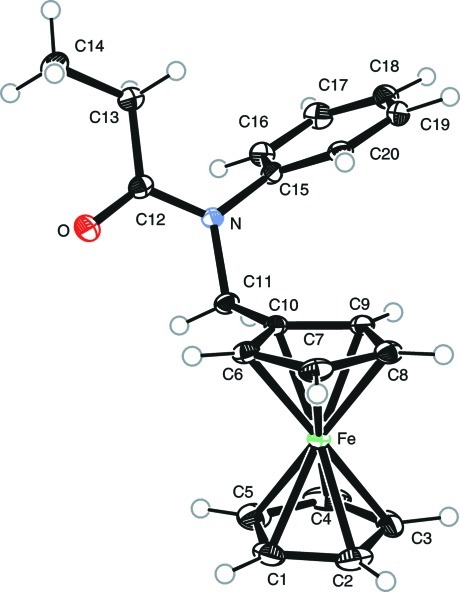
The molecular structure of the title compound, showing the 50% probability displacement ellipsoids.

## supplementary crystallographic information

### Comment

In recent years, the design of new ferrocene derivatives has been of considerable interest, because of their utility in organic synthesis (Cano *et al.*, 1995), asymmetric synthesis (Torres *et al.*, 2002) and medicinal chemistry (Argyropoulos & Coutouli-Argyropoulou, 2002). Our interest in ferrocene derivatives containing *N*-ethyl-*N*-phenylpropionamide group arises from the fact that similar compounds such as *N*-(ferrocenylmethyl)benzene-carboxamide derivatives possess a broad range of biological activities (Kelly *et al.*, 2007). Moreover, ferrocene derivatives that contain *N*-ethyl-*N*-phenylpropionamide moieties are capable of undergoing easy transformation into a variety of functionally useful ferrocenes. The incorporation of *N*-ethyl-*N*-phenylpropionamide in a ferrocene moiety could provide new derivatives with important biological activities since several ferrocene derivatives have already been shown to be active against a number of tumors (Shaabani & Shaghaghi, 2010). Herein, as a continuation of our research related to ferrocenyl derivatives, we report the synthesis and X-ray diffraction characterization of the title compound.

In the title compound (Fig.1), the amide substituent is positioned perpendicular to the plane of the substituted cyclopentadienyl (Cp) ring [the C12—N—C11—C10 torsion angle is 101.3 (2)°]. In the ethanoyl group, the N and O atoms are coplanar [the C11—N—C12—O torsion angle is -0.7 (3)°]. The Fe—C bond distances within the ferrocene group are in a range of 2.041 (3)–2.058 (3) Å for the substituted Cp1 ring (C6–C10) and 2.035 (3)–2.064 (3) Å for the unsubstituted Cp2 ring (C1–C5) (Table 1). The planar Cp rings are nearly parallel to each other [the interplanar angle is 3.7 (1)°]. The Cp rings are essentially staggered and the Fe–centroid distances are 1.659 (3) (Cp1) and 1.652 (3) Å (Cp2). The [Cg1—Fe1—Cg2] angle is 177.60 (2)° (Cg1 and Cg2 are the centroids of the Cp1 and Cp2 rings). Weak C—H···O hydrogen bonds link adjacent molecules (Table 2).

### Experimental

*N*-ferrocenylmethylaniline was obtained according to literature procedures (Osgerby & Pauson, 1961). To a round bottom flask equipped with a reflux condenser and a magnetic stirrer was added under a nitrogen atmosphere a portion of *N*-ferrocenylmethylaniline (6 g, 20 mmol) in 50 ml of anhydride toluene. The resulting suspension was heated at 65°C until total dissolution. 10 ml of propenoique acid was then added and the resulting mixture was vigorously stirred under reflux for 25 min. The reaction mixture was then allowed to cool to room temperature and washed twice with water. The organic layer was then dried and evaporated. The residue was recrystallized from a mixture of ethanol–water to yield *N*-ferrocenymethyl-*N*-phenylpropionamide as yellow-orange needles (yield: 5.85 g, 84%). m.p. 121–122°C. The compounds gave clean ^1^H and ^13^C NMR spectra in CDCl_3_.

### Refinement

H atoms were located from difference Fourier maps and fixed in refinements, with C—H distances in a range of 0.89–1.07 Å and *U*_iso_(H) = 0.02–0.05 Å^2^.

### Crystal data

**Table:**

[Fe(C_5_H_5_)(C_15_H_16_NO)]	*F*(000) = 728
*M**_r_* = 347.23	*D*_x_ = 1.436 Mg m^−^^3^
Monoclinic, *P*2_1_/*n*	Mo *K*α radiation, λ = 0.71073 Å
Hall symbol: -P 2yn	Cell parameters from 3670 reflections
*a* = 13.243 (5) Å	θ = 2–27.5°
*b* = 7.983 (5) Å	µ = 0.94 mm^−^^1^
*c* = 15.248 (5) Å	*T* = 293 K
β = 94.873 (5)°	Needle, orange-yellow
*V* = 1606.2 (13) Å^3^	0.30 × 0.10 × 0.10 mm
*Z* = 4	

### Data collection

**Table:**

Nonius KappaCCD diffractometer	3670 independent reflections
Radiation source: fine-focus sealed tube	2747 reflections with *I* > 2σ(*I*)
Graphite monochromator	*R*_int_ = 0.048
Detector resolution: 9 pixels mm^-1^	θ_max_ = 27.5°, θ_min_ = 2.0°
ω and φ scans	*h* = −17→17
Absorption correction: part of the refinement model (Δ*F*) (*DIFABS*; Walker & Stuart, 1983)	*k* = 0→10
*T*_min_ = 0.823, *T*_max_ = 0.991	*l* = 0→19
16518 measured reflections	

### Refinement

**Table:**

Refinement on *F*^2^	Primary atom site location: structure-invariant direct methods
Least-squares matrix: full	Secondary atom site location: difference Fourier map
*R*[*F*^2^ > 2σ(*F*^2^)] = 0.034	Hydrogen site location: inferred from neighbouring sites
*wR*(*F*^2^) = 0.094	H-atom parameters constrained
*S* = 1.02	*w* = 1/[σ^2^(*F*_o_^2^) + (0.0394*P*)^2^ + 1.6054*P*] where *P* = (*F*_o_^2^ + 2*F*_c_^2^)/3
3670 reflections	(Δ/σ)_max_ = 0.001
209 parameters	Δρ_max_ = 0.40 e Å^−^^3^
0 restraints	Δρ_min_ = −0.38 e Å^−^^3^

### Special details

**Table:**

Geometry. Bond distances, angles *etc*. have been calculated using the rounded fractional coordinates. All su's are estimated from the variances of the (full) variance-covariance matrix. The cell e.s.d.'s are taken into account in the estimation of distances, angles and torsion angles
Refinement. Refinement of *F*^2^ against ALL reflections. The weighted *R*-factor *wR* and goodness of fit *S* are based on *F*^2^, conventional *R*-factors *R* are based on *F*, with *F* set to zero for negative *F*^2^. The threshold expression of *F*^2^ > σ(*F*^2^) is used only for calculating *R*-factors(gt) *etc*. and is not relevant to the choice of reflections for refinement. *R*-factors based on *F*^2^ are statistically about twice as large as those based on *F*, and *R*- factors based on ALL data will be even larger.

### Fractional atomic coordinates and isotropic or equivalent isotropic displacement parameters (Å^2^)

**Table:**

	*x*	*y*	*z*	*U*_iso_*/*U*_eq_	
Fe	−0.07206 (2)	0.49468 (4)	0.32287 (2)	0.0123 (1)	
O	−0.02577 (12)	−0.0369 (2)	0.12252 (12)	0.0221 (5)	
N	0.07871 (14)	0.1884 (2)	0.12666 (12)	0.0143 (5)	
C1	−0.22431 (18)	0.5319 (3)	0.33414 (18)	0.0242 (8)	
C2	−0.1672 (2)	0.6519 (3)	0.38492 (17)	0.0260 (8)	
C3	−0.10882 (19)	0.7428 (3)	0.3266 (2)	0.0328 (9)	
C4	−0.1313 (2)	0.6771 (4)	0.24067 (19)	0.0335 (9)	
C5	−0.2024 (2)	0.5468 (4)	0.24592 (18)	0.0293 (8)	
C6	−0.03618 (18)	0.2442 (3)	0.32216 (16)	0.0165 (7)	
C7	−0.00389 (18)	0.3168 (3)	0.40574 (16)	0.0196 (7)	
C8	0.06553 (18)	0.4483 (3)	0.39123 (15)	0.0184 (7)	
C9	0.07570 (17)	0.4581 (3)	0.29904 (15)	0.0153 (6)	
C10	0.01264 (16)	0.3324 (3)	0.25563 (15)	0.0137 (6)	
C11	−0.00067 (17)	0.2992 (3)	0.15843 (15)	0.0160 (6)	
C12	0.05746 (17)	0.0230 (3)	0.11058 (14)	0.0154 (6)	
C13	0.14207 (18)	−0.0824 (3)	0.07880 (16)	0.0188 (7)	
C14	0.1182 (2)	−0.2690 (3)	0.08018 (18)	0.0237 (8)	
C15	0.17258 (17)	0.2651 (3)	0.10724 (15)	0.0143 (6)	
C16	0.18986 (18)	0.2981 (3)	0.02018 (15)	0.0180 (7)	
C17	0.27898 (19)	0.3783 (3)	0.00165 (17)	0.0220 (8)	
C18	0.34896 (18)	0.4295 (3)	0.06963 (18)	0.0217 (7)	
C19	0.33203 (18)	0.3945 (3)	0.15659 (17)	0.0215 (7)	
C20	0.24406 (17)	0.3113 (3)	0.17543 (15)	0.0173 (7)	
H1	−0.27290	0.44980	0.35450	0.0500*	
H2	−0.16430	0.66850	0.45030	0.0500*	
H3	−0.05810	0.83419	0.34510	0.0500*	
H4	−0.10340	0.70280	0.17850	0.0500*	
H5	−0.23510	0.47710	0.19832	0.0500*	
H6	−0.07780	0.15790	0.31070	0.0500*	
H7	−0.02554	0.28900	0.46350	0.0500*	
H8	0.10160	0.51333	0.43257	0.0500*	
H9	0.11321	0.53210	0.27120	0.0500*	
H11A	0.00230	0.39450	0.12491	0.0500*	
H11B	−0.05942	0.24950	0.14171	0.0500*	
H13A	0.15359	−0.04941	0.01925	0.0226*	
H13B	0.20393	−0.06134	0.11592	0.0226*	
H14A	0.05770	−0.29081	0.04261	0.0356*	
H14B	0.17360	−0.33090	0.05956	0.0356*	
H14C	0.10809	−0.30276	0.13922	0.0356*	
H16	0.14140	0.26610	−0.02292	0.0500*	
H17	0.28780	0.40361	−0.06110	0.0500*	
H18	0.40668	0.50080	0.05460	0.0500*	
H19	0.38560	0.43000	0.20480	0.0500*	
H20	0.23210	0.27879	0.23350	0.0500*	

### Atomic displacement parameters (Å^2^)

**Table:**

	*U*^11^	*U*^22^	*U*^33^	*U*^12^	*U*^13^	*U*^23^
Fe	0.0118 (2)	0.0135 (2)	0.0116 (2)	0.0019 (1)	0.0013 (1)	0.0004 (1)
O	0.0165 (8)	0.0205 (9)	0.0300 (10)	−0.0035 (7)	0.0060 (7)	−0.0006 (7)
N	0.0129 (9)	0.0160 (9)	0.0143 (10)	0.0004 (8)	0.0036 (8)	−0.0038 (8)
C1	0.0145 (11)	0.0209 (13)	0.0377 (15)	0.0037 (10)	0.0057 (11)	0.0052 (11)
C2	0.0261 (13)	0.0341 (15)	0.0174 (12)	0.0161 (12)	−0.0002 (11)	−0.0040 (11)
C3	0.0146 (12)	0.0130 (12)	0.069 (2)	0.0048 (10)	−0.0075 (13)	−0.0013 (12)
C4	0.0311 (15)	0.0396 (16)	0.0318 (16)	0.0216 (14)	0.0142 (13)	0.0215 (13)
C5	0.0228 (13)	0.0378 (16)	0.0254 (14)	0.0126 (12)	−0.0083 (11)	−0.0062 (11)
C6	0.0175 (11)	0.0130 (11)	0.0195 (12)	0.0039 (9)	0.0038 (9)	0.0015 (9)
C7	0.0223 (12)	0.0212 (12)	0.0156 (12)	0.0100 (10)	0.0026 (10)	0.0040 (10)
C8	0.0162 (11)	0.0226 (12)	0.0155 (11)	0.0071 (10)	−0.0034 (9)	−0.0022 (9)
C9	0.0130 (10)	0.0165 (11)	0.0165 (11)	0.0033 (9)	0.0016 (9)	−0.0022 (9)
C10	0.0120 (10)	0.0146 (11)	0.0145 (11)	0.0028 (9)	0.0012 (9)	−0.0006 (9)
C11	0.0163 (11)	0.0182 (11)	0.0136 (11)	0.0033 (9)	0.0017 (9)	−0.0034 (9)
C12	0.0185 (11)	0.0168 (12)	0.0109 (10)	0.0022 (9)	0.0008 (9)	−0.0013 (9)
C13	0.0183 (12)	0.0174 (12)	0.0209 (12)	0.0022 (10)	0.0027 (10)	−0.0011 (10)
C14	0.0262 (13)	0.0160 (12)	0.0287 (14)	0.0026 (11)	0.0008 (11)	−0.0014 (10)
C15	0.0154 (11)	0.0126 (11)	0.0150 (11)	0.0002 (9)	0.0027 (9)	−0.0009 (8)
C16	0.0184 (12)	0.0202 (12)	0.0152 (11)	0.0012 (10)	0.0008 (10)	0.0003 (9)
C17	0.0227 (13)	0.0232 (13)	0.0207 (13)	0.0009 (11)	0.0052 (11)	0.0046 (10)
C18	0.0164 (12)	0.0190 (12)	0.0302 (14)	−0.0017 (10)	0.0054 (11)	−0.0008 (10)
C19	0.0172 (12)	0.0221 (13)	0.0247 (13)	0.0008 (10)	−0.0015 (10)	−0.0086 (10)
C20	0.0159 (11)	0.0201 (12)	0.0160 (12)	0.0031 (10)	0.0025 (9)	−0.0036 (9)

### Geometric parameters (Å, º)

**Table:**

Fe—C1	2.060 (3)	C15—C16	1.391 (3)
Fe—C2	2.064 (3)	C15—C20	1.395 (3)
Fe—C3	2.042 (3)	C16—C17	1.393 (4)
Fe—C4	2.035 (3)	C17—C18	1.392 (4)
Fe—C5	2.046 (3)	C18—C19	1.392 (4)
Fe—C6	2.055 (3)	C19—C20	1.392 (3)
Fe—C7	2.058 (3)	C1—H1	0.9900
Fe—C8	2.055 (3)	C2—H2	1.0000
Fe—C9	2.041 (3)	C3—H3	1.0200
Fe—C10	2.045 (3)	C4—H4	1.0700
O—C12	1.229 (3)	C5—H5	0.9900
N—C11	1.486 (3)	C6—H6	0.8900
N—C12	1.368 (3)	C7—H7	0.9700
N—C15	1.439 (3)	C8—H8	0.9200
C1—C2	1.411 (4)	C9—H9	0.9000
C1—C5	1.405 (4)	C11—H11A	0.9200
C2—C3	1.425 (4)	C11—H11B	0.8900
C3—C4	1.419 (4)	C13—H13A	0.9700
C4—C5	1.410 (4)	C13—H13B	0.9700
C6—C7	1.432 (3)	C14—H14A	0.9600
C6—C10	1.433 (3)	C14—H14B	0.9600
C7—C8	1.426 (3)	C14—H14C	0.9600
C8—C9	1.426 (3)	C16—H16	0.9200
C9—C10	1.431 (3)	C17—H17	0.9900
C10—C11	1.502 (3)	C18—H18	1.0000
C12—C13	1.514 (3)	C19—H19	1.0200
C13—C14	1.523 (4)	C20—H20	0.9500
			
C1—Fe—C2	40.00 (10)	C8—C9—C10	108.5 (2)
C1—Fe—C3	67.68 (10)	Fe—C10—C6	69.93 (13)
C1—Fe—C4	67.62 (10)	Fe—C10—C9	69.36 (13)
C1—Fe—C5	40.03 (11)	Fe—C10—C11	125.68 (16)
C1—Fe—C6	111.65 (10)	C6—C10—C9	107.3 (2)
C1—Fe—C7	115.44 (10)	C6—C10—C11	126.1 (2)
C1—Fe—C8	144.68 (10)	C9—C10—C11	126.5 (2)
C1—Fe—C9	174.56 (10)	N—C11—C10	113.56 (18)
C1—Fe—C10	135.70 (10)	O—C12—N	121.5 (2)
C2—Fe—C3	40.62 (10)	O—C12—C13	121.9 (2)
C2—Fe—C4	68.20 (11)	N—C12—C13	116.6 (2)
C2—Fe—C5	67.73 (11)	C12—C13—C14	112.2 (2)
C2—Fe—C6	138.10 (10)	N—C15—C16	119.5 (2)
C2—Fe—C7	113.03 (10)	N—C15—C20	120.2 (2)
C2—Fe—C8	115.25 (10)	C16—C15—C20	120.3 (2)
C2—Fe—C9	143.16 (10)	C15—C16—C17	119.5 (2)
C2—Fe—C10	175.61 (10)	C16—C17—C18	120.4 (2)
C3—Fe—C4	40.75 (12)	C17—C18—C19	120.0 (2)
C3—Fe—C5	68.10 (12)	C18—C19—C20	119.9 (2)
C3—Fe—C6	178.60 (11)	C15—C20—C19	120.0 (2)
C3—Fe—C7	138.23 (11)	Fe—C1—H1	127.00
C3—Fe—C8	111.35 (10)	C2—C1—H1	128.00
C3—Fe—C9	112.23 (10)	C5—C1—H1	123.00
C3—Fe—C10	140.42 (10)	Fe—C2—H2	124.00
C4—Fe—C5	40.43 (12)	C1—C2—H2	127.00
C4—Fe—C6	140.37 (11)	C3—C2—H2	125.00
C4—Fe—C7	176.63 (10)	Fe—C3—H3	123.00
C4—Fe—C8	136.09 (10)	C2—C3—H3	125.00
C4—Fe—C9	108.54 (10)	C4—C3—H3	127.00
C4—Fe—C10	110.11 (10)	Fe—C4—H4	123.00
C5—Fe—C6	112.26 (11)	C3—C4—H4	134.00
C5—Fe—C7	142.86 (11)	C5—C4—H4	118.00
C5—Fe—C8	175.12 (10)	Fe—C5—H5	127.00
C5—Fe—C9	134.58 (10)	C1—C5—H5	123.00
C5—Fe—C10	108.26 (10)	C4—C5—H5	129.00
C6—Fe—C7	40.74 (9)	Fe—C6—H6	128.00
C6—Fe—C8	68.41 (10)	C7—C6—H6	128.00
C6—Fe—C9	68.56 (10)	C10—C6—H6	123.00
C6—Fe—C10	40.91 (9)	Fe—C7—H7	124.00
C7—Fe—C8	40.57 (9)	C6—C7—H7	129.00
C7—Fe—C9	68.50 (9)	C8—C7—H7	123.00
C7—Fe—C10	68.89 (9)	Fe—C8—H8	129.00
C8—Fe—C9	40.74 (9)	C7—C8—H8	128.00
C8—Fe—C10	68.88 (9)	C9—C8—H8	124.00
C9—Fe—C10	41.01 (9)	Fe—C9—H9	124.00
C11—N—C12	119.50 (18)	C8—C9—H9	127.00
C11—N—C15	117.50 (17)	C10—C9—H9	124.00
C12—N—C15	122.79 (18)	N—C11—H11A	104.00
Fe—C1—C2	70.16 (14)	N—C11—H11B	105.00
Fe—C1—C5	69.45 (15)	C10—C11—H11A	113.00
C2—C1—C5	108.9 (2)	C10—C11—H11B	113.00
Fe—C2—C1	69.83 (14)	H11A—C11—H11B	107.00
Fe—C2—C3	68.84 (14)	C12—C13—H13A	109.00
C1—C2—C3	107.3 (2)	C12—C13—H13B	109.00
Fe—C3—C2	70.53 (14)	C14—C13—H13A	109.00
Fe—C3—C4	69.36 (16)	C14—C13—H13B	109.00
C2—C3—C4	107.8 (2)	H13A—C13—H13B	108.00
Fe—C4—C3	69.89 (16)	C13—C14—H14A	109.00
Fe—C4—C5	70.21 (17)	C13—C14—H14B	109.00
C3—C4—C5	108.0 (2)	C13—C14—H14C	109.00
Fe—C5—C1	70.53 (15)	H14A—C14—H14B	109.00
Fe—C5—C4	69.37 (16)	H14A—C14—H14C	110.00
C1—C5—C4	108.1 (2)	H14B—C14—H14C	109.00
Fe—C6—C7	69.74 (14)	C15—C16—H16	118.00
Fe—C6—C10	69.16 (13)	C17—C16—H16	123.00
C7—C6—C10	108.2 (2)	C16—C17—H17	117.00
Fe—C7—C6	69.53 (13)	C18—C17—H17	122.00
Fe—C7—C8	69.58 (13)	C17—C18—H18	118.00
C6—C7—C8	107.9 (2)	C19—C18—H18	122.00
Fe—C8—C7	69.85 (13)	C18—C19—H19	118.00
Fe—C8—C9	69.13 (13)	C20—C19—H19	122.00
C7—C8—C9	108.0 (2)	C15—C20—H20	118.00
Fe—C9—C8	70.13 (13)	C19—C20—H20	122.00
Fe—C9—C10	69.64 (13)		
			
C2—Fe—C1—C5	120.1 (2)	C3—Fe—C8—C9	−99.70 (16)
C3—Fe—C1—C2	−38.08 (16)	C4—Fe—C8—C7	−179.30 (16)
C3—Fe—C1—C5	82.06 (18)	C4—Fe—C8—C9	−59.8 (2)
C4—Fe—C1—C2	−82.30 (17)	C6—Fe—C8—C7	−37.74 (14)
C4—Fe—C1—C5	37.84 (18)	C6—Fe—C8—C9	81.79 (15)
C5—Fe—C1—C2	−120.1 (2)	C7—Fe—C8—C9	119.5 (2)
C6—Fe—C1—C2	140.59 (15)	C9—Fe—C8—C7	−119.5 (2)
C6—Fe—C1—C5	−99.27 (18)	C10—Fe—C8—C7	−81.81 (14)
C7—Fe—C1—C2	96.15 (16)	C10—Fe—C8—C9	37.71 (14)
C7—Fe—C1—C5	−143.71 (17)	C2—Fe—C9—C8	62.7 (2)
C8—Fe—C1—C2	57.7 (2)	C2—Fe—C9—C10	−177.75 (16)
C8—Fe—C1—C5	177.88 (18)	C3—Fe—C9—C8	97.37 (16)
C10—Fe—C1—C2	−178.64 (15)	C3—Fe—C9—C10	−143.04 (15)
C10—Fe—C1—C5	−58.5 (2)	C4—Fe—C9—C8	140.80 (15)
C1—Fe—C2—C3	−118.8 (2)	C4—Fe—C9—C10	−99.61 (15)
C3—Fe—C2—C1	118.8 (2)	C5—Fe—C9—C8	177.98 (16)
C4—Fe—C2—C1	80.71 (17)	C5—Fe—C9—C10	−62.4 (2)
C4—Fe—C2—C3	−38.09 (16)	C6—Fe—C9—C8	−81.39 (15)
C5—Fe—C2—C1	36.94 (16)	C6—Fe—C9—C10	38.21 (14)
C5—Fe—C2—C3	−81.86 (17)	C7—Fe—C9—C8	−37.46 (14)
C6—Fe—C2—C1	−62.1 (2)	C7—Fe—C9—C10	82.13 (14)
C6—Fe—C2—C3	179.13 (16)	C8—Fe—C9—C10	119.6 (2)
C7—Fe—C2—C1	−102.69 (16)	C10—Fe—C9—C8	−119.6 (2)
C7—Fe—C2—C3	138.51 (16)	C1—Fe—C10—C6	−67.93 (19)
C8—Fe—C2—C1	−147.28 (15)	C1—Fe—C10—C9	173.60 (15)
C8—Fe—C2—C3	93.92 (17)	C1—Fe—C10—C11	52.8 (3)
C9—Fe—C2—C1	172.86 (16)	C3—Fe—C10—C6	179.35 (16)
C9—Fe—C2—C3	54.1 (2)	C3—Fe—C10—C9	60.9 (2)
C1—Fe—C3—C2	37.51 (15)	C3—Fe—C10—C11	−60.0 (3)
C1—Fe—C3—C4	−81.13 (17)	C4—Fe—C10—C6	−146.08 (14)
C2—Fe—C3—C4	−118.7 (2)	C4—Fe—C10—C9	95.46 (15)
C4—Fe—C3—C2	118.7 (2)	C4—Fe—C10—C11	−25.4 (2)
C5—Fe—C3—C2	80.87 (16)	C5—Fe—C10—C6	−103.20 (15)
C5—Fe—C3—C4	−37.78 (16)	C5—Fe—C10—C9	138.33 (15)
C7—Fe—C3—C2	−66.2 (2)	C5—Fe—C10—C11	17.5 (2)
C7—Fe—C3—C4	175.12 (16)	C6—Fe—C10—C9	−118.47 (19)
C8—Fe—C3—C2	−104.35 (16)	C6—Fe—C10—C11	120.7 (2)
C8—Fe—C3—C4	137.01 (15)	C7—Fe—C10—C6	37.37 (14)
C9—Fe—C3—C2	−148.37 (15)	C7—Fe—C10—C9	−81.10 (14)
C9—Fe—C3—C4	92.99 (16)	C7—Fe—C10—C11	158.1 (2)
C10—Fe—C3—C2	173.37 (15)	C8—Fe—C10—C6	81.00 (14)
C10—Fe—C3—C4	54.7 (2)	C8—Fe—C10—C9	−37.47 (13)
C1—Fe—C4—C3	81.30 (16)	C8—Fe—C10—C11	−158.3 (2)
C1—Fe—C4—C5	−37.47 (16)	C9—Fe—C10—C6	118.47 (19)
C2—Fe—C4—C3	37.98 (15)	C9—Fe—C10—C11	−120.8 (3)
C2—Fe—C4—C5	−80.80 (17)	C12—N—C11—C10	101.3 (2)
C3—Fe—C4—C5	−118.8 (2)	C15—N—C11—C10	−83.8 (2)
C5—Fe—C4—C3	118.8 (2)	C11—N—C12—O	−0.7 (3)
C6—Fe—C4—C3	178.68 (15)	C11—N—C12—C13	−179.74 (19)
C6—Fe—C4—C5	59.9 (2)	C15—N—C12—O	−175.3 (2)
C8—Fe—C4—C3	−66.3 (2)	C15—N—C12—C13	5.6 (3)
C8—Fe—C4—C5	174.90 (16)	C11—N—C15—C16	−101.1 (3)
C9—Fe—C4—C3	−102.82 (16)	C11—N—C15—C20	76.5 (3)
C9—Fe—C4—C5	138.41 (16)	C12—N—C15—C16	73.6 (3)
C10—Fe—C4—C3	−146.36 (15)	C12—N—C15—C20	−108.7 (3)
C10—Fe—C4—C5	94.86 (17)	Fe—C1—C2—C3	58.87 (17)
C1—Fe—C5—C4	119.0 (2)	C5—C1—C2—Fe	−58.83 (19)
C2—Fe—C5—C1	−36.92 (15)	C5—C1—C2—C3	0.0 (3)
C2—Fe—C5—C4	82.07 (18)	Fe—C1—C5—C4	−59.4 (2)
C3—Fe—C5—C1	−80.92 (17)	C2—C1—C5—Fe	59.27 (18)
C3—Fe—C5—C4	38.07 (17)	C2—C1—C5—C4	−0.2 (3)
C4—Fe—C5—C1	−119.0 (2)	Fe—C2—C3—C4	59.60 (18)
C6—Fe—C5—C1	97.62 (17)	C1—C2—C3—Fe	−59.49 (17)
C6—Fe—C5—C4	−143.40 (16)	C1—C2—C3—C4	0.1 (3)
C7—Fe—C5—C1	62.3 (2)	Fe—C3—C4—C5	60.1 (2)
C7—Fe—C5—C4	−178.75 (17)	C2—C3—C4—Fe	−60.34 (18)
C9—Fe—C5—C1	178.93 (14)	C2—C3—C4—C5	−0.2 (3)
C9—Fe—C5—C4	−62.1 (2)	Fe—C4—C5—C1	60.16 (19)
C10—Fe—C5—C1	141.17 (15)	C3—C4—C5—Fe	−59.93 (19)
C10—Fe—C5—C4	−99.85 (17)	C3—C4—C5—C1	0.2 (3)
C1—Fe—C6—C7	−104.32 (15)	Fe—C6—C7—C8	−59.20 (17)
C1—Fe—C6—C10	135.86 (14)	C10—C6—C7—Fe	58.61 (16)
C2—Fe—C6—C7	−66.7 (2)	C10—C6—C7—C8	−0.6 (3)
C2—Fe—C6—C10	173.53 (15)	Fe—C6—C10—C9	59.52 (16)
C4—Fe—C6—C7	175.06 (16)	Fe—C6—C10—C11	−120.1 (2)
C4—Fe—C6—C10	55.2 (2)	C7—C6—C10—Fe	−58.97 (16)
C5—Fe—C6—C7	−147.63 (14)	C7—C6—C10—C9	0.6 (3)
C5—Fe—C6—C10	92.56 (15)	C7—C6—C10—C11	−179.1 (2)
C7—Fe—C6—C10	−119.82 (19)	Fe—C7—C8—C9	−58.77 (16)
C8—Fe—C6—C7	37.58 (14)	C6—C7—C8—Fe	59.16 (17)
C8—Fe—C6—C10	−82.23 (14)	C6—C7—C8—C9	0.4 (3)
C9—Fe—C6—C7	81.53 (15)	Fe—C8—C9—C10	−59.27 (16)
C9—Fe—C6—C10	−38.29 (13)	C7—C8—C9—Fe	59.22 (17)
C10—Fe—C6—C7	119.82 (19)	C7—C8—C9—C10	−0.1 (3)
C1—Fe—C7—C6	94.23 (15)	Fe—C9—C10—C6	−59.88 (16)
C1—Fe—C7—C8	−146.47 (14)	Fe—C9—C10—C11	119.8 (2)
C2—Fe—C7—C6	138.22 (14)	C8—C9—C10—Fe	59.58 (16)
C2—Fe—C7—C8	−102.48 (15)	C8—C9—C10—C6	−0.3 (3)
C3—Fe—C7—C6	178.57 (15)	C8—C9—C10—C11	179.4 (2)
C3—Fe—C7—C8	−62.1 (2)	Fe—C10—C11—N	173.98 (15)
C5—Fe—C7—C6	55.2 (2)	C6—C10—C11—N	−96.0 (3)
C5—Fe—C7—C8	174.45 (17)	C9—C10—C11—N	84.5 (3)
C6—Fe—C7—C8	119.30 (19)	O—C12—C13—C14	−10.3 (3)
C8—Fe—C7—C6	−119.30 (19)	N—C12—C13—C14	168.8 (2)
C9—Fe—C7—C6	−81.69 (15)	N—C15—C16—C17	177.5 (2)
C9—Fe—C7—C8	37.62 (14)	C20—C15—C16—C17	−0.2 (4)
C10—Fe—C7—C6	−37.52 (14)	N—C15—C20—C19	−176.1 (2)
C10—Fe—C7—C8	81.78 (14)	C16—C15—C20—C19	1.5 (4)
C1—Fe—C8—C7	59.6 (2)	C15—C16—C17—C18	−1.9 (4)
C1—Fe—C8—C9	179.14 (16)	C16—C17—C18—C19	2.7 (4)
C2—Fe—C8—C7	96.56 (15)	C17—C18—C19—C20	−1.3 (4)
C2—Fe—C8—C9	−143.92 (14)	C18—C19—C20—C15	−0.8 (4)
C3—Fe—C8—C7	140.78 (15)		

### Hydrogen-bond geometry (Å, º)

**Table:**

*D*—H···*A*	*D*—H	H···*A*	*D*···*A*	*D*—H···*A*
C4—H4···O^i^	1.07	2.50	3.292 (4)	130

Symmetry code: (i) *x*, *y*+1, *z*.
